# Cue Weighting in Perception of the Retroflex and Non-Retroflex Laterals in the Zibo Dialect of Chinese

**DOI:** 10.3390/bs13060469

**Published:** 2023-06-04

**Authors:** Bing Dong, Jie Liang, Chang Liu

**Affiliations:** 1 Department of English, School of Foreign Languages, Tongji University, Shanghai 200092, China; dongbingtj@tongji.edu.cn (B.D.); liangjie56@163.net (J.L.); 2 Department of Speech, Language, and Hearing Sciences, The University of Texas at Austin, Austin, TX 78712, USA

**Keywords:** retroflex and non-retroflex laterals, the schwa following the laterals, Zibo dialect, F1, consonant-to-vowel (C/V) duration ratio, cue weighting

## Abstract

This study investigated cue weighting in the perception of the retroflex and non-retroflex lateral contrast in the monosyllabic words /ɭə/ and /lə/ in the Zibo dialect of Chinese. A binary forced-choice identification task was carried out among 32 natives, using computer-modified natural speech situated in a two-dimensional acoustic space. The results showed that both acoustic cues had a significant main effect on lateral identification, with F1 of the following schwa being the primary cue and the consonant-tos-vowel (C/V) duration ratio as a secondary cue. No interaction effect was found between these two acoustic cues. Moreover, the results indicated that acoustic cues were not equally weighted in production and perception of the syllables /ɭə/ and /lə/ in the Zibo dialect. Future studies are suggested involving other acoustic cues (e.g., the F1 of laterals) or adding noise in the identification task to better understand listeners’ listening strategies in their perception of the two laterals in the Zibo dialect.

## 1. Introduction

Laterals are common in languages across the world, existing in about 82% of the 317 sample languages in the UPSID (UCLA Phonological Segment Inventory Database). Among these laterals, about 87% are produced in the dental/alveolar region, while the retroflex lateral only appears in 6.7% of them [1]. The comparative rarity of the phonemic retroflex lateral vs. dental/alveolar lateral contrast has motivated research on different languages, for example the Dravidian languages of India, the indigenous languages of Australia and several dialects of Chinese.

Research on the retroflex and non-retroflex lateral contrast in the Dravidian languages spoken in South Asia (e.g., India, Pakistan, Sri Lanka) includes articulatory and acoustic studies of Tamil [2,3,4], Malayalam [5,6,7] and Kannada [7]. The articulatory studies on the retroflex and non-retroflex lateral contrast in the Dravidian languages found that there was a dental/alveolar vs. retroflex lateral contrast with a subapical articulation of the retroflex lateral in Tamil [2,3,4], Malayalam [5,6,7] and Kannada [7], while previous studies of the Australian indigenous languages Arrernte, Pitjantjatjara and Warlpiri have showed that the retroflex lateral had an apical articulation [8,9].

In addition to articulation measures, acoustic features such as F1, F2 and F3 as well as the duration of laterals have also been examined in previous studies. No consistency was found in the F1 or F2 frequencies between the retroflex and non-retroflex laterals in the Dravidian languages [2,3,4,5,6,7]. Similarly, in the Australian indigenous languages Arrernte, Pitjantjatjara and Warlpiri, no significant difference was found in the F1 or F2 between the two laterals [9,10,11]. As for F3, there was a consistently lower F3 in the retroflex lateral /ɭ/ than its non-retroflex counterpart /l/ in Tamil, Malayalam and Kannada as well as in the Australian indigenous languages Arrernte, Pitjantjatjara and Warlpiri. Moreover, the duration of the retroflex lateral /ɭ/ in the Dravidian languages mentioned above was found to be shorter than that of the non-retroflex /l/. For the three Australian indigenous languages, Arrernte, Pitjantjatjara and Warlpiri, no significant difference was found in duration between the two laterals. In sum, a significantly lower F3 in /ɭ/ than in /l/ was found in the Dravidian languages and the three Australian indigenous languages, which was also considered the main correlate of retroflexes in previous studies of vowels and consonants [12,13,14].

Aside from the articulatory and acoustic characteristics of the retroflex lateral, the phonotactics of /ɭ/ and /l/ have also been discussed in previous research. The occurrence of retroflexes within a syllable and a prosodic word is cross-linguistically asymmetrical. In general, retroflex consonants in CV structure were neutralized or not as well distinguished as in VC structure [8,14,15,16]. In both the Dravidian languages of India and the indigenous languages of Australia, the retroflex lateral /ɭ/ was found to frequently occur intervocalically, less often in the word-final position and rarely in the word-initial position [2,7,8,9,10,11].

Although a number of studies have investigated retroflex and non-retroflex laterals in multiple languages, little research has been carried out on the production and perception of the two laterals in the sound systems of Chinese dialects, such as the Zibo dialect, which are unique and different from other languages. The retroflex and non-retroflex lateral contrast (/ɭ/ vs. /l/) in the Zibo dialect of Chinese differed in both its acoustic and phonotactic characteristics from the lateral contrast in other languages of the world. Therefore, these two laterals in the Zibo dialect were selected as the speech stimuli in this study of lateral perception.

According to the phonology of the Zibo dialect, the retroflex lateral can only appear in the syllable-initial position followed by an atypical schwa, which is higher and only appears following the retroflex lateral /ɭ/ as in /ɭə/, while the non-retroflex lateral /l/ also only appears in the syllable-initial position, but can be followed by most vowels in the Zibo dialect, including monophthongs, diphthongs and triphthongs [17].

In addition, acoustic studies have indicated that the F1 frequency of /ɭ/ in the Zibo dialect is slightly but significantly lower than that of /l/, while no significant difference for F2 and F3 frequencies was found between the two laterals [18,19], consistent with the findings of research on laterals in several other dialects of Chinese [20,21]. Interestingly, the formant structures of the schwa following the two laterals in the Zibo dialect showed similarities and differences. That is, the F1 frequency of the schwa following /ɭ/ was significantly lower than that following /l/ with little speaker variability, while there was no significant difference in F2 frequency of the following vowels [18,19]. The F3 frequency for the schwa following /ɭ/ was significantly higher than that for the schwa following /l/, but the difference was small with large speaker variability [19]. Moreover, the duration of the retroflex lateral /ɭ/ was significantly longer than the non-retroflex lateral /l/, and the duration of the schwa following /ɭ/ was significantly shorter than that following /l/. Therefore, the retroflex lateral /ɭ/ had a significantly larger consonant-to-vowel (C/V) duration ratio in the monosyllabic word /ɭə/ than the non-retroflex lateral /l/ in /lə/. These acoustic measures indicated that the F1 frequency of the schwa following the lateral and the C/V duration ratio were two primary acoustic production cues separating the two laterals in the Zibo dialect and were selected for acoustic manipulation for the perceptual measures in this study.

The C/V duration ratio, defined as the consonant closure duration divided by the preceding vowel duration, is a primary acoustic cue for voicing in several western languages such as English and German [22,23,24,25,26]. For example, it plays a critical role in English voicing in syllable-final and intervocalic positions, e.g., his vs. hiss and dibber vs. dipper, when other cues such as closure voicing and aspiration are ambiguous [22,23]. It is also employed as a voicing cue in speech production and perception for several other Germanic languages, such as Swedish and German [24,25,26]. On the other hand, the role of the C/V duration ratio in the identification of laterals is still unknown, although acoustic studies have indicated its importance in separating the retroflex and non-retroflex laterals in production in the Zibo dialect [18,19]. In comparison with absolute durations of the vowel and consonant segments that are highly dependent on speaking rate, the C/V duration ratio, largely independent of speaking rate, is a significantly more powerful and consistent cue for phonological voicing in English [23]. Thus, in the present study, the C/V duration ratio rather than the absolute duration of speech segments was selected as a primary acoustic factor to manipulate. Since the laterals in the Zibo dialect only appear in the initial position of a CV structure, the C/V duration ratio in this study is defined as the duration ratio of the lateral with respect to the following vowels, as in the monosyllabic words /ɭə/ and /lə/.

Overall, the two laterals in the Zibo dialect are unique due to its phonological structure (e.g., only at the syllable initial position) and the significant differences shown in two acoustic cues: F1 frequency of the schwa following the laterals and the C/V duration ratio of laterals. However, relatively few studies have been performed on the perception of retroflex vs. non-retroflex laterals, which contrast in a sound system such as Zibo Chinese. Thus, this study was to investigate how the two important acoustic cues affected perception of the words /ɭə/ and /lə/ in the Zibo dialect. The two acoustic cues were manipulated systematically at five levels each, resulting in a total of 25 stimuli. Two research questions were proposed: first, whether and how each acoustic cue influenced the identification of the two laterals; and second, what the perceptual weight of each acoustic cue was, e.g., the role of the other acoustic cue when one acoustic cue was ambiguous.

## 2. Methods

### 2.1. Participants

A group of 32 Zibo natives (17 females and 15 males) took part in this study. All participants were aged between 25 and 43 years old, with normal hearing and speaking ability, who communicated in the Zibo dialect in their daily lives. Listeners were paid for their participation.

### 2.2. Stimuli

As mentioned above, in addition to the F1 frequency of the following vowel and the C/V duration cue, the two laterals also differed in the F1 frequency by themselves. A pilot experiment with four young native speakers of the Zibo dialect suggested that the schwa following the lateral was dominant to determine the identification of laterals. That is, the identification of a hybrid stimulus (e.g., the initial /ɭ/ mixed with the /ə_2_/ following /l/ to generate /ɭə_2_/ or the initial /l/ mixed with the /ə_1_/ following /ɭ/ to generate /lə_1_/) was determined by the following vowel rather than the initial lateral. For example, /ɭə_2_/ was identified as /lə_2_/ 100%, while/lə_1_/was perceived as /ɭə_1_/ 100%. Thus, in this study, only two acoustic cues—the F1 frequency of the following vowel and C/V duration ratio—were manipulated, but not the F1 frequency of the initial lateral.

All stimuli were computer-modified natural speech situated in a two-dimensional acoustic space. The original audio materials used in this study were extracted from the natural utterances of one female native speaker (aged 36), recorded in a soundproof recording booth using a Sennheiser GSP 602 headset microphone at a sampling rate of 44.1 kHz. The word list for this study contained 80 compound words with the two laterals in the Zibo dialect, namely 40 words each for /ɭə/ and /lə/. The monosyllabic words /ɭə/ and /lə/ in the word list had the same tone: a low falling tone with a tone value of 31 in the Zibo dialect, which is the most frequent citation tone where /ɭə/ and /lə/ appear in this dialect. They were recorded by the female speaker with a Chinese carrier sentence /və tu [compound] tə ɭə sã piã/ or /və tu [compound] tə lə sã piã/ (English meaning: “I read /ɭə/ as in [compound] three times” or “I read /lə/ as in [compound] three times”). The carrier sentence was designed to help the speaker pronounce more naturally [27], with a sentence focus on the monosyllabic word /ɭə/ or /lə/ (the second /ɭə/ or /lə/ in the carrier sentence). The monosyllabic words /ɭə/ and /lə/, rather than those in the compounds, were extracted for stimulus generation. Altogether 40 monosyllabic /ɭə/ words and 40 monosyllabic /lə/ words were recorded and extracted from the carrier sentence. Based on the judgement of two phoneticians, one /ɭə/ and one /lə/ were selected from the recordings that were clearly pronounced and had no creakiness in the syllable pronunciation. The two syllables were equalized in duration, room-mean-square level and pitch contour, and then used as the standard signal to synthesize speech stimuli with the manipulation of the F1 frequency of the following schwa and the C/V duration ratio in Praat [28].

Since the lateral and the following schwa have a one-to-one match in the two words, two sets of speech stimuli with systematic changes in the two acoustic cues were generated in this study: one set based on /ɭə_1_/ and the other set based on /lə_2_/. Here, [ə_1_] and [ə_2_] are used respectively to distinguish between the schwas following /ɭ/ and /l/ for ease of readability.

In the first set of stimuli, the standard signal /ɭə_1_/ (with equal duration, intensity, and pitch contour with the standard signal /lə_2_/ used for the synthesis of the second set of stimuli) was used to generate the speech stimuli. As shown in Table 1, first, the F1 value of the vowel segment [ə_1_] in /ɭə_1_/ was manipulated at five levels based on the average F1 values of 40 tokens of [ə_1_] and 40 tokens of [ə_2_] from the recording, e.g., 661 Hz for [ə_1_] and 840 Hz for [ə_2_]. The five F1 frequencies were equally distant on the auditory Bark scale. Second, the C/V duration ratio continuum was also created at five levels with the average C/V duration ratio of the 40 recordings of /ɭə_1_/ as level 1 and the average C/V duration ratio of the 40 recordings of /lə_2_/ as level 5. For the speech synthesis, except for the changes in the two acoustic cues manipulated above, other acoustic features such as the F2 and F3 frequencies were not changed. Altogether, there were a total of 25 different stimuli (5 levels on the F1 frequency × 5 levels on the C/V duration ratio) for the first set of speech stimuli, which used /ɭə_1_/ as the standard signal.

Likewise, another set of speech stimuli with similar manipulations of the two acoustic cues, e.g., five levels on the F1 of the following schwa and five levels on the C/V duration ratio, were generated with the syllable /lə_2_/ as the standard signal. Altogether, 50 different stimuli (25 stimuli generated from /ɭə_1_/ and 25 stimuli generated from /lə_2_/) were synthesized in the study. In sum, for the two sets of stimuli, only the F1 frequency of the following schwa and the C/V duration ratio of the lateral were changed systematically, with all other acoustic features unchanged.

The perception experiment was carried out in a quiet room on a computer with Sennheiser headphones (Model 280 Pro) using ExperimentMFC implemented in Praat. Subjects were required to perform a binary forced-choice word identification task in which they clicked on one of two Chinese characters presented on the computer screen, i.e., /ɭə/ with the corresponding Chinese character meaning “two” or /lə/ with the corresponding Chinese character meaning “hot”, in a self-paced and self-selected fashion after hearing a speech signal. Once listeners responded, the next trial was automatically started. The listeners had up to three chances to listen to a single stimulus with no response time limit. For each listener, the 50 speech stimuli were presented in a random order in one block and a similar block was repeated three times, resulting in a total of 150 stimulus presentations.

The formal experiment of each participant was preceded by an eight-trial practice session to familiarize the subjects with the procedure. No feedback was provided in the practice or test sessions.

### 2.3. Data Analysis

Data collected from Praat were separated into two groups: data for 75 stimuli generated from /ɭə_1_/ (5 formant levels × 5 duration ratio levels × 3 repetitions) and data for 75 stimuli generated from /lə_2_/ (5 formant levels × 5 duration ratio levels × 3 repetitions). The data were analyzed separately in order to see the results of perception with one acoustic cue in the “lateral-schwa” structure fixed and the other cue changing along the continuum. A mixed-effects logistic regression model was used for the stimuli of each group to quantify the use of the two cues simultaneously by modeling how well category affiliation was predicted by the F1 value of the following schwa or the C/V duration ratio in the syllable. The logistic regression model tests for significant main effects of the two cues and interaction between cues were used to evaluate differences in cue weight across conditions [29]. The general logistic regression equation for the present identification task is given as the following formula:

log(odds (/ɭə/)) = ln(p(/ɭə/)/p(/lə/)) = α + β_F1_ × F1 value of schwa + β_DR_ × the C/V duration ratio of lateral + β_Interaction_ × (F1 value of schwa×the C/V duration ratio of lateral)

In this equation, α is the intercept of the regression model. The coefficients (βs) of the logistic regression model quantify the impact of a one-step difference in one of the cues on the log odds of a subject’s response. Morrison [30] has suggested that these coefficients can be interpreted as a measure of a subject’s reliance on each cue. In other words, the magnitude of the coefficient reflects the degree to which subjects use a specific cue in making their responses.

## 3. Results

### 3.1. Results for Stimuli Generated from /ɭə_1_/

**Figure 1:**
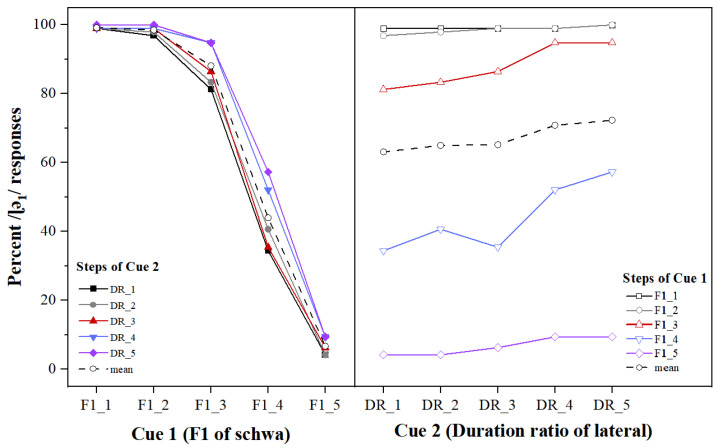
Percentages of/ɭə_1_/response as a function of Cue 1 (**left** panel) and Cue 2 (**right** panel) for stimuli generated from /ɭə_1_/, with colored solid lines representing five different levels and the black dashed line representing the mean.

In the response percentages results for /ɭə_1_/ on different levels of Cue 1 (F1 value of the following schwa), as expected, the subjects’ judgments vary systematically with the increase in the F1 value of the following schwa (from 661 Hz in step 1 to 840 Hz in step 5), as seen in the left panel of Figure 1. Generally, the /ɭə_1_/ response curve to stimuli generated from /ɭə_1_/ tends to decrease with Cue 1 changing from level 1 to level 5, which suggests that Cue 1 has a large effect on participants’ responses to the stimuli. That is, the lower the F1 value of the following schwa, the more likely they are identified by the participants as /ɭə_1_/.

In terms of the results for percent /ɭə_1_/ responses for different levels of Cue 2, i.e., the C/V duration ratio in the syllable “lateral-schwa”, the average percentage of /ɭə_1_/ response increased gradually from step 1 to step 5. As seen in the right panel of Figure 1, the average percentage of /ɭə_1_/ responses increased gradually at the beginning from step 1 at 63.13% to 72.29% at step 5, which indicates a general tendency that for all stimuli on different levels of Cue 2, the percentage of /ɭə_1_/ responses tends to increase with Cue 2 changing from level 1 to level 5. Generally, the results suggest that for the stimuli in this identification task, the larger the C/V duration ratio, the more likely the participants identified the stimuli as /ɭə_1_/.

To evaluate the effects of the two cues on the identification of the monosyllabic words /ɭə/ and /lə/, a mixed-effects logistic regression model was used to analyze the data. Cue 1 and Cue 2 were set as fixed effects and Participant and Stimuli were set as random effects. The analysis of the model shows that the marginal R^2^ is 0.754 and the conditional R^2^ is 0.872, indicating that 75.4% of the variance is caused by the independent effects in the model (Cue 1 and Cue 2), 11.8% of the variance is due to the random effects (Participant and Stimuli) and the model can explain a total of 87.2% of the variance. The results of the mixed-effects logistic regression model are shown in Table 2.

Results show that first, there is a significant main effect of Cue 1 (χ^2^(1) = 434.07, *p* < 0.001), indicating that an increase in the F1 frequency steps of the following schwa decreases /ɭə/ responses; and second, there is a significant main effect of Cue 2 (χ^2^(1) = 30.96, *p* < 0.001), indicating that an increase in the steps of the C/V duration ratio increases /ɭə/ responses. No significant interaction effect was found between Cue 1 and Cue 2 (*p* = 0.080).

These results suggest that subjects used both cues in their identification of the stimuli. The coefficients of the logistic regression analysis show to what extent a one-step difference in one of the cues caused a change in the log odds of a participant’s response of /ɭə_1_/. As for cue weighting, Cue 1 (β = −3.17) is the primary cue for identification of the syllable /ɭə/ and Cue 2 (β = 0.50) is the secondary perceptual cue, suggesting that Zibo natives weight the F1 value of the following schwa much more heavily than the C/V duration ratio in the syllable on their identification of /ɭə/ and /lə/.

### 3.2. Results for Stimuli Generated from /lə_2_/

**Figure 2:**
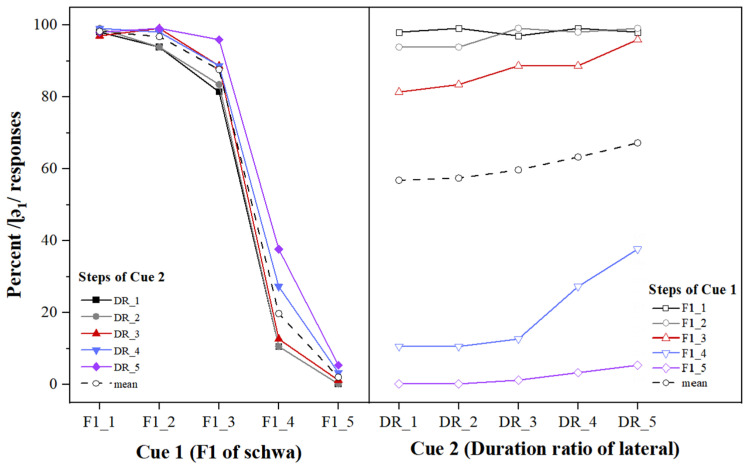
Percentages of /ɭə_1_/ response as a function of Cue 1 (**left** panel) and Cue 2 (**right** panel) for stimuli generated from /lə_2_/, with colored solid lines representing five different levels and the black dashed line representing the mean.

For the results of response percentages for /lə_2_/ on different levels of Cue 1, the subjects’ judgments varied with the increase in the F1 value of the following schwa, as shown in the left panel of Figure 2.

These systematic decreases show that participants relied on Cue 1 heavily in their responses to the stimuli. For stimuli on different Cue 1 levels, generally the lower the F1 frequency of the following schwa is, the higher the percentage of /ɭə_1_/ response is, which is identical to the result for stimuli in the first set. For the results for percentage of /ɭə_1_/ responses on different levels of Cue 2, the average percentage of /ɭə_1_/ response increased systematically from 56.67% to 67.08% from step 1 to step 5 of the C/V duration ratio, as seen in the right panel of Figure 2.

A mixed-effects logistic regression model was used to evaluate the effect of the two cues on the identification of /ɭə/ and /lə/ for this group of stimuli. Similarly, Cue 1 and Cue 2 were set as fixed effects, and Participant and Stimuli were set as random effects. The analysis of the model shows that the marginal R^2^ is 0.720 and the conditional R^2^ is 0.880, showing that 72% of the variance is caused by the independent effects in the model (Cue 1 and Cue 2), 16% of the variance is due to the random effects (Participant and Stimuli) and the model can explain a total of 88% of the variance. The results of the mixed-effects logistic regression model are shown in Table 3.

The results show that first, there is a significant main effect of Cue 1 (χ^2^(1) = 168.06, *p* < 0.001), indicating that an increase in the steps of Cue 1 decreases /ɭə/ responses; and second, there is a significant main effect of Cue 2 (χ^2^(1) =6.73, *p* = 0.009), indicating that an increase in the steps of Cue 2 increases /ɭə/ responses. No significant interaction effect was found between Cue 1 and Cue 2.

These results indicate that subjects use both of the two cues in their identification of the stimuli of this set. Cue 1 (β = −3.07) is the primary cue for the identification of the syllable /ɭə/ and Cue 2 (β = 0.51) is the secondary perceptual cue, suggesting that Zibo natives weight the F1 value of the following schwa much more heavily than the C/V duration ratio in the identification of the two laterals, which echoes the findings in Section 3.1.

## 4. Discussion

The purpose of this study was to assess how the two acoustic cues, i.e., the F1 frequency of the following schwa and the consonant-to-vowel duration ratio, affected the perception of the two laterals in the monosyllabic words /ɭə_1_/ and /lə_2_/. The two acoustic cues were systematically manipulated to generate two sets of speech stimuli, one originated from /ɭ/ and the other originated from/l/.

### 4.1. Cue Weighting of the Two Acoustic Cues in the Perception of /ɭə/ and /lə/

For the two sets of stimuli, both the F1 of the following schwa and the C/V duration ratio in the monosyllabic words had a significant main effect on the identification of the two laterals. That is, the lower the F1 of the following schwa, the greater the possibility it was identified as /ɭə_1_/, while the higher the F1 of the following schwa, the higher possibility it was identified as /lə_2_/. As for the C/V duration ratio of the lateral in the “lateral-schwa” syllable, in general, the larger the duration ratio of the lateral in the syllable was, the more likely it was identified as /ɭə_1_/; the smaller the C/V duration ratio of the lateral in the syllable was, the more likely it was identified as /lə_2_/. No interaction effect was found between the two acoustic cues in either set of stimuli. The results from the mixed effects logistic regression model showed that the β of the F1 of the following schwa was much larger than that of the C/V duration ratio, suggesting that the F1 frequency of the following schwa was the primary cue, while the C/V duration ratio played a secondary role in the identification of the two laterals. In particular, as shown in Figure 1 and Figure 2, when the F1 frequency of the following schwa dominated phonetic identification, the change in the C/V duration ratio had little effect on the identification of the two laterals, also indicating the critical role of the F1 frequency of the following schwa.

In fact, spectral information as the primary cue with phonetic duration as the secondary cue has also been found in a number of previous studies. For example, F1 and F2 frequencies are the primary cues for English vowel perception, while vowel duration plays a secondary role for native speakers [31,32]. In the present study, since duration is not a distinctive phonemic feature in the Zibo dialect, the effect of speech duration may be even smaller, if any.

As mentioned previously, the C/V duration ratio was used in the present study to reflect the temporal information of the lateral in relation to that of the following schwa in the monosyllabic word. When the spectral information was ambiguous (e.g., see the curves of Cue 1 at level 3 and 4 in Figure 1 and Figure 2), this temporal information significantly affected the identification as a secondary cue. This result is similar to the finding in Port and Dalby [23] on the role of the C/V duration ratio in the perception of voiced and voiceless consonants in the intervocalic and final positions, in which although the C/V duration ratio might be dominated by non-temporal cues, it showed a significant impact on the perception of syllable-final voicing in English when other acoustic features were held constant and yet ambiguous.

### 4.2. Production and Perception Link of Laterals in the Zibo Dialect

Contrasts for speech sounds differ along multiple phonetic dimensions and phonetic cue weighting can be quantified in the context of both production and perception. Research examining the relative alignment of cue weighting across modalities has revealed both parallels and asymmetries between the modalities [29]. For example, English speakers’ productions of syllable-initial /b/ and /p/ showed large and consistent differences in VOT. In addition, /b/ was followed by a lower f0 than /p/ on average, but such differences were much smaller and less consistent than those in VOT [33,34]. This difference in cue weighting between the two cues in production is also reflected in perception: when asked to categorize sounds varying in the duration of aspiration and f0, subjects’ responses mainly depended on aspiration (the primary cue), with f0 in a secondary but detectable role [35]. This is an example of a match of both cue use and cue weighting between production and perception of a phonemic contrast. Languages differ in the use of phonetic cues and the relative importance given to these cues. Cue use in perception tends to reflect community production norms on a broad level; however, research work directly comparing individual use across modalities has revealed matches and mismatches in cue use between perception and production. Shultz et al. [36] explored the connections between production and perception by investigating the manner in which native English speakers’ relative weighting of VOT corresponded to that of onset f0 in both production and perception of English syllable-initial consonant voicing. Their finding showed a significant negative correlation of VOT and onset f0 in production, and the coefficients suggested that all the participants were primarily users of VOT, with some participants putting remarkable weight on onset f0 and other participants not using onset f0 in their production. However, in perception, no statistically significant results were found for the correlation between the two cues, although with a positive trend. These results indicated that the relative weighting of acoustic cues might be processed differently in production and perception of the same phonemic contrast.

This study also showed both a match and a mismatch in acoustic cue use between the production and perception of /ɭə_1_/ and /lə_2_/ in the Zibo dialect. The two important cues that showed large and consistent differences between /ɭə_1_/ and /lə_2_/ in production [18,19], i.e., the F1 of the following schwa and the C/V duration ratio of the laterals, were also important in perception. However, native speakers’ production of /ɭə_1_/and /lə_2_/ showed large and consistent differences in both the F1 value of the following schwa and the C/V duration ratio of the lateral, while in perception, participants’ responses were mainly determined by the F1 value of the following schwa (the primary cue), with the C/V duration ratio as a secondary cue. This suggests that the two cues differed in their importance in production and perception, showing a mismatch of cue weighting between these two modalities. In speech production, speakers aim to produce an integrated acoustic property to instantiate the contrast between two speech units while compensating for idiosyncratic variation [36]. In contrast, in perception, people may use one cue primarily with other cues as secondary. The asymmetry in cue weighting between the production and perception of Zibo laterals may be associated with the different strategies people use during speech production and perception.

### 4.3. Limitation and Future Studies

It is worth mentioning that the percentages of /ɭə_1_/ responses in both groups of stimuli were over chance rate (50% in this study), showing a tendency of more /ɭə_1_/ responses than /lə_2_/ in both sets of stimuli. There are two possible reasons. First, this result is possibly related to the word frequency effect in word processing, in which high-frequency words are processed more efficiently than low-frequency words [37,38]; secondly, when listeners are unsure of what they have heard, they are more likely to report hearing a high-frequency lexical item than a low-frequency one [39]. Therefore, the word exposure difference might lead to the favorable response bias toward /ɭə_1_/ in identification due to its higher frequency than /lə_2_/ when used both independently and in compound words in the daily life of Zibo natives. Second, the higher /ɭə_1_/ responses for stimuli based on /ɭə_1_/ than for stimuli based on /lə_2_/ also indicates that aside from the F1 frequency of the following schwa as well as the C/V duration ratio of the lateral in the syllable, other acoustic cues, such as the F1 frequency of the laterals and the higher formants of laterals and schwas, may also affect the identification of the syllables. In addition, the manipulation of lateral and vowel durations in this study may result in a change in the naturalness of formant transition, and the effect of such a change needs further investigation in future study.

During speech perception, listeners must decide which cues are relevant and determine the relative importance of each cue as well as integrate other signal-external cues [29]. The C/V duration ratio of the lateral may seem much less important than the formant cue in the identification of the two Zibo laterals, but it may facilitate successful perception of speech under adverse listening conditions, e.g., in the presence of interfering noise and when listeners have non-native linguistic backgrounds or hearing impairments. In future studies, stimuli involving other acoustic cues (e.g., a lower F1 in the retroflex lateral) in different listening conditions (e.g., quiet and noisy) can be used to investigate listeners’ cue-weighting strategies in their perception of the two laterals in the Zibo dialect in a variety of listening environments.

## 5. Conclusions

In the present study, two acoustic cues, the F1 of the schwa following the lateral and the C/V duration ratio, were systematically manipulated to investigate their roles in the identification of two laterals: /ɭ/ and /l/ in the Zibo dialect in Chinese. Both acoustic cues showed significant effects on lateral perception. Moreover, listeners relied on the F1 of the following schwa more heavily than the C/V duration ratio in the syllable. These results suggest that although both cues were presented consistently by native speakers in their production, listeners had different listening strategies in cue weighting for their perception.

## Figures and Tables

**Table 1:** Levels of F1 value of [ə_1_] (Cue 1) and levels of the consonant-to-vowel (C/V) duration ratio of the lateral (Cue 2) of the stimuli.

(a)					
Levels of Cue 1	F1_1	F1_2	F1_3	F1_4	F1_5
F1 of /ə/ (Hz)	661	702	746	791	840
(b)					
Levels of Cue 2	DR_1	DR_2	DR_3	DR_4	DR_5
C/V duration ratio	1.36	1.64	1.92	2.20	2.48
Duration of C (ms)	131	139	147	154	162
Duration of V (ms)	96	88	80	73	65

**Table 2:** Coefficients of the two cues in the mixed-effects logistic regression model for stimuli generated from /ɭə_1_/.

Predictor: Fixed Effects	β	SE	Exp (B)	z	*p*
Intercept	2.78	0.34	16.16	8.27	<0.001
Cue 1 (F1)	−3.17	0.15	0.04	−20.83	<0.001
Cue 2 (C/V)	0.50	0.09	1.65	5.56	<0.001
Cue 1 × Cue 2	−0.13	0.08	0.88	−1.75	0.080

**Table 3:** Coefficients of the two cues in the mixed-effects logistic regression model for stimuli generated from /lə_2_/.

Predictor: Fixed Effects	β	SE	Exp (B)	z	*p*
Intercept	1.18	0.41	3.27	2.88	0.004
Cue 1	−3.07	0.24	0.05	−12.96	<0.001
Cue 2	0.51	0.20	1.66	2.59	0.009
Cue 1×Cue 2	0.17	0.15	1.19	1.11	0.266

## Data Availability

The data presented in this study are available on request from the corresponding author.
